# Exploring how the sustainability of water and sanitation projects influences community health outcomes in Butambala District, Uganda

**DOI:** 10.1108/IJHCQA-02-2026-0049

**Published:** 2026-11-05

**Authors:** Namiyingo Sophia, Lunga Msengana

**Affiliations:** University of South Africa, Pretoria, South Africa; Department of Business Leadership, University of South Africa, Pretoria, South Africa

**Keywords:** Water and sanitation, Sustainability, Public health outcomes, SDG 6, SDG 3, Rural Uganda

## Abstract

**Purpose:**

This study explores how the sustainability of water and sanitation projects influences community health outcomes in rural Uganda.

**Design/methodology/approach:**

A qualitative design was adopted, involving in-depth interviews with 18 purposively selected participants at saturation, including caregivers, LC1 chairpersons, water committee members, borehole caretakers and village health team members. Data were analysed thematically to identify patterns linking sustainability dynamics to hygiene practices and disease exposure.

**Findings:**

Findings reveal that infrastructure breakdowns directly increase health risks, while weak leadership, limited accountability and low social trust undermine maintenance and community commitment. Coping strategies often involve health trade-offs, and environmental stress further intensifies system fragility. Strong participatory governance and community ownership were found to strengthen both sustainability and preventive health behaviours.

**Practical implications:**

Practically, the study highlights the need for integrated governance reforms, climate-responsive planning, transparent financial management and strengthened community ownership mechanisms to safeguard health outcomes. The findings suggest that water infrastructure investments alone are insufficient without sustained institutional support and adaptive capacity building.

**Originality/value:**

The study contributes an integrated sustainability–health perspective, demonstrating that achieving safe water and sanitation outcomes requires not only infrastructure provision but also accountable governance, social cohesion and climate-responsive management in rural contexts.

## Introduction

Access to clean water and adequate sanitation services is a fundamental human need and a cornerstone of global sustainable development agendas (Kyambade *et al.*, 2025). The United Nations Sustainable Development Goal 6 (SDG 6) aims to ensure availability and sustainable management of water and sanitation (W&S) for all by 2030, emphasising that improvements in water supply and sanitation systems are critical for public health, economic productivity, and environmental sustainability. Globally, billions of people remain without safely managed W&S services, and this deficit continues to drive high rates of preventable diseases such as diarrhoeal illnesses, particularly in low- and middle-income countries (LMICs) where infrastructure challenges and service reliability issues are most acute (Kyambade *et al.*, 2026a). Studies have shown that without sustained access to safe W&S, communities remain vulnerable to waterborne and hygiene-related diseases, undermining not only household health but also broader development outcomes aligned with Sustainable Development Goal 3 (SDG 3) - good health and well-being (Kyambade *et al.*, 2026b; Priyadarshini and Gundimeda, 2025).

In Uganda, significant investments have been made to expand W&S infrastructure in both urban and rural areas, yet substantial challenges persist in ensuring these systems remain functional and health-promoting over time (Kyambade *et al.*, 2026a). National assessments confirm that service levels for safely managed W&S remain low, with persistent gaps in reliability and functionality that disproportionately affect rural communities and informal settlements (Nakibuule *et al.*, 2026). Beyond access, the sustainability of water, sanitation, and hygiene (WASH) projects meaning their continued operation, maintenance, and long-term support, has increasingly been recognised as a key determinant of whether health gains are maintained or eroded after project implementation ends (Kyambade *et al.*, 2026b). In many contexts, projects initially raise service availability but fail to sustain technical, institutional, and social elements needed to prevent a return to unsafe conditions, thereby limiting their contribution to sustained improvements in community health.

The literature on W&S sustainability highlights that long-term success is not solely a technical engineering issue, but reflects complex interactions among community engagement, governance, financial mechanisms, and environmental conditions (Kyambade *et al.*, 2026a). Analyses of rural W&S projects reveal that communities with more inclusive decision-making and active participation tend to maintain higher levels of functionality and health benefits, while systems lacking supportive governance and local ownership degrade more rapidly after initial project completion (Chimphero *et al.*, 2026). Similarly, evaluation studies in various LMIC settings suggest that sustainability is influenced by initial planning processes, the capacity to manage and maintain water systems, and the degree to which health benefits are integrated into programme objectives (Simion Luduşanu *et al.*, 2025). However, even when projects appear successful in physical terms, the health outcomes associated with sustained access such as reduced diarrhoeal incidence and improved hygiene behaviours, are seldom tracked beyond the project lifespan.

Despite this growing body of research, a critical knowledge gap remains in understanding how the sustainability of W&S projects not just their existence, shapes the everyday health experiences and coping strategies of rural communities (Kyambade *et al.*, 2026c; Win *et al.*, 2024; Nchofoung *et al.*, 2024). Many existing studies focus on infrastructure metrics (e.g. borehole functionality, latrine presence) or broad health statistics, but fewer provide rich, contextual insight into the lived experiences of households and local leaders as they negotiate the breakdowns, maintenance challenges, and health risks associated with unsustainable systems. Without such qualitative understanding, policy and practice risk overemphasising initial service delivery while overlooking the social and behavioural mechanisms that determine whether health gains such as reduced diarrhoeal disease risk are actually realised and sustained in rural settings.

Unlike much of the existing WASH research in Uganda and Sub-Saharan Africa, which has largely emphasised infrastructure functionality, service access, community participation, or aggregate health outcomes (Win *et al.*, 2024; Nchofoung *et al.*, 2024), this study integrates technical, governance, and economic sustainability to explain how the continued functioning of rural WASH projects shapes community health experiences. By qualitatively examining the pathways through which these interconnected sustainability conditions influence health outcomes in Butambala District, the study provides a contextually grounded and multidimensional contribution that extends beyond the predominantly infrastructure- and access-focused perspectives of previous research.

This study therefore seeks to fill this gap by adopting a qualitative case approach to explore the lived realities of W&S project sustainability and its implications for community health in Uganda's Butambala District. Guided by this broader objective, the research is centred on the following question: How does the sustainability of W&S projects influence community health outcomes in Butambala District, Uganda?

## Literature review

### SDG 6 and SDG 3: the Water–Health nexus

W&S are fundamental to public health, with safely managed services advancing SDG 6 and reducing preventable morbidity and mortality under SDG 3 (Kyambade *et al.*, 2026c). Unsafe water, inadequate sanitation, and poor hygiene remain major contributors to diarrhoeal diseases, particularly in low- and middle-income countries (Prüss-Ustün *et al.*, 2019). Evidence shows that reliable water and sanitation services significantly reduce diarrhoeal risks (Wolf *et al.*, 2018), while integrated improvements in water supply, sanitation, and hygiene are more effective than isolated interventions (Fewtrell *et al.*, 2005). These findings highlight that sustained service functionality and consistent use, rather than infrastructure provision alone, are essential for translating SDG 6 investments into lasting SDG 3 health outcomes.

### Conceptualising sustainability in W&S projects

Within WASH scholarship, sustainability has shifted from infrastructure provision to long-term service continuity (Kyambade *et al.*, 2025), encompassing technical, financial, social, institutional, and environmental dimensions (Montgomery *et al.*, 2009; Benavides-Muñoz, 2025). Sustained services require post-construction support, effective governance, and maintenance systems beyond community self-management (Lockwood and Smits, 2011), particularly in Sub-Saharan Africa, where infrastructure failures, financial constraints, and weak oversight undermine continuity (Chowns, 2015). Despite inconsistent conceptualisations, this study defines W&S project sustainability as the continuous delivery of safe, reliable, and socially acceptable services through three interconnected dimensions: technical functionality, involving infrastructure reliability and maintenance (Chowns, 2015; Whaley and Cleaver, 2017); governance effectiveness, encompassing leadership, accountability, institutional support, and stakeholder coordination (Mansuri and Rao, 2013; Whaley and Cleaver, 2017); and economic viability, involving adequate financing for operations, maintenance, and replacement (Montgomery *et al.*, 2009; Lockwood and Smits, 2011). Together, these dimensions underpin sustained service delivery and community health outcomes.

### Community-based management (CBM) and governance challenges

CBM remains central to rural water governance in Sub-Saharan Africa, despite persistent institutional limitations. Whaley and Cleaver (2017) argue that infrastructure functionality alone overlooks power relations, accountability gaps, and institutional fragmentation, while water committees often lack adequate authority, resources, and technical capacity. Similarly, Chowns (2015) attributes prolonged service failures to weak governance, unclear institutional responsibilities, limited district support, and supply chain disruptions rather than community unwillingness. These weaknesses undermine trust, financial contributions, and maintenance, highlighting the importance of accountable leadership and institutional coordination for sustained services and public health protection.

### Participation, leadership, and sustainability outcomes

Stakeholder participation strengthens W&S sustainability when supported by meaningful engagement, transparency, accountability, and inclusive decision-making rather than symbolic involvement (Mansuri and Rao, 2013). Effective leadership builds community trust, mobilises financial contributions, and coordinates maintenance, enhancing project continuity (Kyambade *et al.*, 2025). Conversely, exclusionary decisions and financial opacity weaken community commitment and increase infrastructure vulnerability. Thus, participatory governance and accountable leadership are essential for sustaining W&S services and protecting community health.

### Sustainability and health outcomes: the missing qualitative link

Although improved WASH services are associated with reduced disease burden (Wolf *et al.*, 2018; Prüss-Ustün *et al.*, 2019), limited qualitative research explains how sustainability failures influence lived health experiences, coping behaviours, and disease vulnerability. Existing studies emphasise infrastructure functionality and health indicators while giving insufficient attention to governance, maintenance, financial constraints, and community practices (Whaley and Cleaver, 2017; Benavides-Muñoz, 2025). In Sub-Saharan Africa, these challenges reflect broader institutional and accountability weaknesses (Chowns, 2015). Particularly in rural Uganda, limited context-specific evidence explains how these interconnected factors shape health outcomes. This study addresses this gap by examining how technical, governance, and economic dimensions of WASH sustainability influence community health experiences, coping strategies, and disease risks, contributing to integrated SDG 6–SDG 3 implementation.

## Methodology

### Research design

This study adopted a qualitative explanatory case study design to examine how W&S project sustainability influences community health outcomes in Butambala District, Uganda. The qualitative approach enabled an in-depth exploration of lived experiences, governance dynamics, service reliability, and health-related coping behaviours beyond quantitative indicators (Creswell and Poth, 2016). The case study design facilitated contextual understanding of interconnected infrastructure, institutional, and health conditions (Yin, 2018), while its explanatory orientation explored how and why sustainability challenges influence disease vulnerability, exposure risks, and preventive health practices.

W&S project sustainability was operationalised as the capacity of WASH interventions to continuously provide reliable, functional, and acceptable services beyond implementation through three interconnected dimensions. Technical sustainability encompassed infrastructure functionality, service reliability, maintenance, and repair systems; governance sustainability addressed leadership, accountability, stakeholder participation, institutional coordination, and community ownership; while economic sustainability concerned financial arrangements and resource mobilisation for continued operation and maintenance. This conceptualisation aligns with WASH scholarship emphasising that long-term service continuity depends on integrated technical, institutional, and financial systems (Montgomery *et al.*, 2009; Lockwood and Smits, 2011).

### Study area

The study was conducted in Butambala District, Uganda, a predominantly rural district where W&S projects operate largely under CBM arrangements. The district experiences recurring challenges such as borehole breakdowns, shared sanitation facilities, limited maintenance capacity, and seasonal water stress. These contextual features provided a relevant setting for examining how sustainability conditions surrounding W&S projects influence community health outcomes within the broader SDG 6 and SDG 3 framework.

### Study population and sampling

The study population comprised stakeholders directly involved in or affected by W&S projects in Butambala District, Uganda. Participants included household members, particularly caregivers responsible for water collection and hygiene practices, Local Council I (LC1) chairpersons, water user committee members, borehole caretakers or technicians, and Village Health Team (VHT) members. These groups were selected for their practical knowledge of infrastructure functionality, maintenance, governance, and associated health risks. The study employed purposive sampling to identify information-rich participants capable of providing detailed contextual insights rather than statistically generalisable findings (Patton, 2015). Maximum variation sampling was incorporated to capture diverse experiences across stakeholder categories, sub-counties, leadership structures, environmental conditions, and project performance levels (see Table 1).

**Table 1:** Distribution of participants across stakeholder categories

Stakeholder category	Role/experience	Number of participants
Household representatives/caregivers	Users of W&S services; health and hygiene experiences	6
Local Council I (LC1) leaders	Community governance and accountability roles	3
Water user committee members	Project management and maintenance responsibilities	4
Borehole caretakers/technicians	Technical functionality and repair experiences	2
VHT members	Community health monitoring and disease prevention perspectives	3
*Total*		*18*

**Source(s):** Authors’ own work

Data collection and analysis proceeded concurrently, allowing emerging insights to inform subsequent interviews. Saturation was assessed through the absence of new codes, repetition of existing categories, and limited emergence of additional conceptual insights concerning sustainability–health relationships. Saturation was achieved after 18 interviews, as subsequent accounts largely reinforced established themes. This approach aligns with Guest *et al.* (2006), who emphasise that saturation occurs when additional interviews yield limited new analytical insights. The sampling strategy enabled comparison across service users, governance actors, technical personnel, and health stakeholders, providing a comprehensive understanding of how sustainability conditions influence community health outcomes.

### Data collection

Data were collected through semi-structured in-depth interviews, allowing participants to describe their lived experiences while enabling flexible probing (Creswell and Poth, 2016). The interview guide explored WASH project reliability and maintenance, community participation, leadership, coping strategies during service disruptions, and perceived health outcomes, particularly diarrhoeal disease risks and hygiene practices. The guide underwent expert review and pilot testing to ensure clarity, content validity, and alignment with study objectives, with feedback informing refinements to question wording and sequencing. Where necessary, interviews were conducted in local languages using translation and back-translation procedures to preserve conceptual equivalence. Interviews took place at convenient locations, were audio-recorded with consent, transcribed verbatim, and translated into English where applicable. Ethical safeguards included informed consent, voluntary participation, the right to withdraw, confidentiality, anonymisation, and secure data storage.

### Data analysis

Data were analysed using Braun and Clarke's (2006) six-phase thematic analysis framework. Transcripts were repeatedly reviewed for familiarisation, followed by inductive coding of meaningful segments reflecting sustainability conditions and health experiences. Codes were systematically compared across transcripts, refined, merged, or separated based on emerging patterns, and grouped into preliminary themes. These themes were iteratively reviewed against coded extracts and the complete dataset, then refined, defined, and named to ensure coherence and accurate representation of participants' experiences. Analytical decisions were documented through coding notes and an audit trail. Although coding was inductive, interpretation was informed by established WASH sustainability dimensions, including technical reliability, financial mechanisms, governance, stakeholder participation, and institutional support (Montgomery *et al.*, 2009; Lockwood and Smits, 2011). Particular attention was given to identifying pathways through which sustainability weaknesses, such as service interruptions and inadequate maintenance, influenced unsafe coping practices and community health risks.

### Trustworthiness and rigour

Methodological rigour was ensured through credibility, transferability, dependability, and confirmability (Lincoln and Guba, 1985). Credibility was strengthened through prolonged participant engagement, iterative probing, and member clarification during interviews, while transferability was supported by detailed descriptions of the study context and participants. Dependability and confirmability were enhanced through an audit trail documenting coding decisions, theme development, and analytical reflections. To minimise researcher bias, preliminary codes and themes underwent peer debriefing and independent verification, with disagreements resolved through consensus. Reflexive notes further ensured that interpretations remained grounded in participants' experiences rather than researcher assumptions.

## Findings

These findings present how W&S project sustainability influences community health outcomes in Butambala District, Uganda. Five interconnected themes revealed how technical, governance, economic, social, and environmental factors interact to shape health risks, coping behaviours, and community well-being. The analysis further identifies how these sustainability dimensions reinforce one another, creating pathways of vulnerability or resilience that influence community health outcomes.

### Theme 1: infrastructure breakdown and direct health exposure

Participants identified infrastructure breakdowns as the most immediate pathway linking WASH sustainability failures to health risks. Borehole malfunctions, delayed repairs, deteriorating sanitation facilities, and unreliable services forced households to rely on potentially contaminated alternatives, increasing exposure to waterborne diseases. As one respondent explained,

When the borehole breaks, we go back to the swamp. That water is not clean, but we have no choice. (Resp_03)

This statement reveals the constrained agency experienced by households when infrastructure becomes unreliable. The phrase “we have no choice” reflects how sustainability gaps remove safe options and normalise risky practices. The shift from protected boreholes to swamp water represents a direct transition from controlled water access to high-contamination exposure. Another participant elaborated on the delays in repair systems, stating,

Sometimes the pump spoils and it can take weeks before anyone comes to fix it. During that time, children start getting diarrhoea. (Resp_11)

This account underscores the temporal dimension of sustainability. The gap between breakdown and repair creates prolonged periods of exposure. The explicit mention of children highlights heightened vulnerability among younger household members, suggesting that service interruptions have unequal health impacts within communities. Water scarcity also affected hygiene behaviours. A caregiver explained,

When water is scarce, we reduce handwashing. You cannot use water for washing when you are not sure of tomorrow. (Resp_07)

This quote illustrates how sustainability challenges extend beyond drinking water to hygiene practices. Under conditions of uncertainty, households prioritise immediate survival needs over preventive hygiene behaviours, inadvertently increasing disease transmission risks. The tension between water conservation and hygiene maintenance reveals how service instability shapes behavioural adaptation. Leadership perspectives further contextualised infrastructure ageing and lack of long-term planning. An LC1 leader observed,

We constructed these facilities years ago, but maintenance was never planned properly. Now they are old and failing. (Resp_02)

This reflection highlights structural weaknesses in post-construction planning. Infrastructure longevity was compromised not necessarily by initial construction quality alone but by the absence of systematic maintenance strategies. Sustainability, therefore, was undermined by governance and planning gaps rather than simple community neglect. Health surveillance actors also confirmed the relationship between infrastructure breakdown and illness patterns. A VHT member stated,

Cases of stomach infections rise when the boreholes fail because people use shallow wells. (Resp_14)

This observation connects service reliability directly to observable health outcomes at community level. The link between shallow well use and stomach infections reinforces the exposure pathway created by sustainability failures. Trust in infrastructure also emerged as a critical factor influencing behaviour. A water committee member remarked,

People stop trusting the borehole if it keeps breaking down. They prefer the spring because it is always there. (Resp_05)

This statement reflects the erosion of confidence in formal infrastructure when reliability declines. Even if a spring is less safe, its consistency becomes more valued than intermittent safe infrastructure. Thus, reliability influences behavioural preference and risk perception. Sanitation infrastructure was equally vulnerable to environmental and structural deterioration. One respondent explained,

The latrine collapsed during heavy rains. Since then, some families go to the bush. (Resp_18)

This highlights the fragility of sanitation systems under climatic stress. The collapse of latrines leads to open defecation practices, increasing environmental contamination and disease transmission pathways.

The findings reveal that infrastructure failures translate governance and maintenance weaknesses into health risks. Inadequate planning, delayed repairs, and limited resources disrupt services, forcing communities toward unsafe alternatives that compromise hygiene, erode trust, and increase disease exposure. Thus, infrastructure instability reflects broader institutional weaknesses, highlighting the need for effective governance, timely maintenance, and adequate financing to sustain WASH services, prevent disease, and protect SDG 6 health benefits.

### Theme 2: coping strategies and risk trade-offs

When W&S services became unreliable, communities adopted coping strategies such as rainwater harvesting, alternative water sources, rationing, and storage. Although these responses demonstrated resilience and adaptability, they often compromised hygiene and safety, increasing contamination and disease risks. As one participant explained,

We started harvesting rainwater, but the tanks are not covered properly, so sometimes insects fall inside. (Resp_09)

This account illustrates adaptive behaviour in response to unreliable boreholes, yet it also exposes new contamination pathways. While rainwater harvesting represents an effort to reduce dependence on failing infrastructure, inadequate storage conditions undermine its safety. The absence of proper covers and filtration mechanisms introduces microbial and environmental contaminants, demonstrating how adaptation under weak sustainability conditions can reproduce health risks. Another respondent noted,

If the borehole is crowded, some people fetch from open wells because they cannot wait for hours. (Resp_01)

Here, time becomes a critical constraint shaping risk decisions. Long queues at functional boreholes often caused by breakdowns elsewhere, push households toward less safe options. The decision to use open wells is not framed as ignorance of risk but as a pragmatic response to competing daily demands. This reflects how sustainability challenges reshape risk perception and normalise compromise. Water rationing practices further reveal how scarcity alters hygiene behaviour. A mother shared,

When there is no water, we prioritise drinking. Bathing and cleaning are reduced. (Resp_12)

This statement underscores the hierarchy of needs within water-scarce contexts. Drinking water becomes the priority, while hygiene practices such as handwashing, bathing, and cleaning utensils are reduced. Such behavioural shifts weaken preventive health barriers and increase exposure to communicable diseases. The trade-off between conservation and cleanliness reflects how sustainability failures constrain protective health behaviours. Water storage emerged as another coping mechanism with potential risks. A caretaker explained,

People store water for many days. Sometimes it changes colour, but they still use it. (Resp_04)

Prolonged storage without safe containment increases the likelihood of contamination. Yet households continue using stored water despite visible quality changes, reflecting limited alternatives. This illustrates how sustainability instability forces households into calculated acceptance of degraded quality rather than complete deprivation. Economic constraints also shaped coping strategies. A VHT member observed,

Some households boil water, but when charcoal is expensive, they stop. (Resp_15)

This comment reveals how preventive practices are contingent upon financial resources. Boiling water, an important protective measure depends on access to fuel. When fuel costs rise, households suspend treatment practices, increasing exposure risk. Sustainability, therefore, is not only infrastructural but also economically mediated. Cultural familiarity and habituation further influenced behaviour. An elderly respondent remarked,

We know the swamp water is not safe, but we grew up using it, so we continue. (Resp_17)

This highlights how long-standing practices and normalised exposure shape health behaviour. When infrastructure reliability declines, communities may revert to historically familiar water sources, even when aware of contamination risks. The interplay between tradition, convenience, and constrained choice complicates sustainability–health dynamics. Environmental pressures intensified these coping mechanisms. One interviewee explained,

During drought, everyone struggles. Even the rain tanks dry up. (Resp_06)

This illustrates how climate variability compounds infrastructure fragility. When drought reduces groundwater yield and rainwater storage simultaneously, coping options narrow further, amplifying vulnerability. Sustainability weaknesses and environmental stress interact to intensify health risks.

The findings show that community coping strategies reflect both resilience and vulnerability, as households adapt to unreliable WASH services amid inadequate infrastructure, financial constraints, and weak institutional support. Rather than behavioural neglect, these short-term adaptations reflect limited choices that may compromise hygiene and increase health risks. Sustainable W&S services therefore require reliable infrastructure and institutional support to prevent households from sacrificing safety for access, time, or affordability.

### Theme 3: leadership, governance, and accountability gaps

Leadership and governance emerged as critical determinants of W&S sustainability and community health. Participants highlighted weak oversight, unclear responsibilities, fragmented coordination, and financial opacity, which undermined infrastructure reliability, community trust, participation, and long-term commitment to safe services. As one respondent explained,

We don’t know who is responsible when the borehole breaks. The committee blames the district, and the district blames us. (Resp_08)

This statement illustrates a classic diffusion of responsibility, where overlapping institutional roles create ambiguity rather than clarity. In the absence of clearly defined mandates and enforcement mechanisms, maintenance failures remain unresolved. When breakdowns occur, blame-shifting replaces action, prolonging service interruptions and increasing health exposure risks. Such fragmentation weakens institutional resilience and undermines coordinated responses to infrastructure failure. Leadership visibility and consistency were also questioned. Another participant observed:

Leaders call meetings only when there is a problem. After that, they disappear. (Resp_10)

This comment reflects reactive rather than proactive governance. Leadership engagement appears crisis-driven, activated only during visible breakdowns, and not sustained through continuous monitoring or preventive planning. The absence of routine oversight and follow-up mechanisms weakens maintenance culture and reduces community confidence in leadership structures. Financial accountability emerged as a particularly sensitive issue affecting stakeholder commitment. A community member explained:

We contribute money, but we never see reports. That makes people stop contributing. (Resp_05)

Here, sustainability is directly linked to transparency. When financial contributions are not accompanied by visible reporting or accountability mechanisms, trust deteriorates. Reduced willingness to contribute subsequently constrains repair funds, creating a cycle where governance gaps produce financial instability, which then further undermines infrastructure reliability and health protection. Even local leaders acknowledged institutional limitations. An LC1 chairperson admitted:

Sometimes we lack support from higher authorities. We try, but without technical help, we fail. (Resp_02)

This statement highlights vertical governance challenges. While community-level leadership exists, its effectiveness is constrained by limited technical support, insufficient supervision from district authorities, and weak institutional backing. The decentralisation of responsibility without adequate capacity building or resource allocation creates governance gaps that affect both sustainability and service quality. External stakeholder disengagement also contributed to accountability weaknesses. One water user remarked:

The NGO promised follow-up visits, but they never returned. (Resp_13)

This quote reflects the discontinuity of partner engagement. Initial project implementation often generates optimism, but when external actors withdraw without structured transition mechanisms, communities are left without technical or advisory support. The absence of sustained partnership arrangements weakens continuity and reinforces perceptions of abandonment. Health actors further emphasised the limits of isolated interventions. A VHT member explained:

Health education alone is not enough if the water source is broken. (Resp_14)

This insight underscores the interdependence between governance and health outcomes. Behavioural change campaigns and hygiene education lose effectiveness when structural systems fail. Without reliable water infrastructure, health promotion efforts cannot translate into sustained preventive behaviour, demonstrating that governance stability is foundational for public health protection. Ultimately, participants summarised the governance dilemma succinctly:

Without clear leadership, the project becomes everyone’s responsibility and no one’s responsibility. (Resp_16)

This conclusion captures the systemic nature of accountability failure. When roles are unclear and coordination mechanisms weak, collective responsibility dissolves into institutional inertia. The project exists in name but lacks functional stewardship.

The findings reveal that WASH sustainability failures stem from interconnected governance weaknesses, including poor accountability, reactive leadership, financial opacity, and fragmented coordination. These challenges delay maintenance, erode trust, weaken participation and resource contributions, and disrupt services, increasing reliance on unsafe alternatives and disease vulnerability. Sustainable WASH services therefore require clear institutional responsibilities, accountable leadership, transparent financial management, effective oversight, and sustained multi-level support to protect community health.

### Theme 4: social trust, participation, and health protection

Social trust and community participation emerged as essential to W&S sustainability and health protection. Participants indicated that cohesion, transparency, and collective ownership strengthened facility maintenance and hygiene practices, while mistrust, exclusion, and participation fatigue weakened both. These findings highlight the importance of social relationships and participatory governance beyond technical and financial considerations. As one participant explained,

When we work together to clean around the borehole, the place stays safe. (Resp_05)

This quote illustrates how cooperative maintenance strengthens environmental hygiene and reduces contamination risks. Joint cleaning activities not only preserve infrastructure functionality but also foster shared responsibility. The physical act of working together reinforces social bonds, creating informal monitoring systems that protect water quality and promote hygienic surroundings. However, not all communities demonstrated such cohesion. Another respondent observed:

Only a few people volunteer for maintenance. The rest just complain. (Resp_01)

This reflects uneven participation and the burden placed on a small, committed minority. When responsibility is concentrated among a few individuals, maintenance becomes unsustainable over time. Such imbalances create resentment and fatigue, eventually reducing overall engagement and weakening the protective systems necessary for safe water access. Transparency and leadership integrity were repeatedly linked to community willingness to participate. A respondent noted:

If leaders are transparent, people are willing to contribute for repairs. (Resp_07)

This statement underscores the relationship between governance trust and financial sustainability. When community members perceive accountability in decision-making and resource management, they are more inclined to contribute financially and physically to maintenance efforts. Trust thus acts as a catalyst for collective investment in sustainability. Gender dynamics also influenced participation patterns. A mother explained:

Women fetch water every day, but men control the money for repairs. (Resp_11)

This comment highlights a structural imbalance between labour contribution and financial decision-making. Women, as primary water users and managers of household hygiene, carry the daily operational burden, yet they may have limited authority over financial resources. Such gendered governance gaps can weaken inclusive decision-making and reduce responsiveness to practical W&S needs. Inclusivity in meetings and governance processes also shaped engagement levels. One participant remarked:

When meetings are not inclusive, people stop attending. (Resp_04)

Exclusion whether through scheduling, communication, or dominance by particular groups, discourages participation and erodes collective ownership. When stakeholders feel unheard or marginalised, they withdraw, diminishing the community's ability to sustain infrastructure collaboratively. Youth exclusion further weakened sustainability potential. Another interviewee added:

Youth are rarely involved, yet they are strong enough to help maintain facilities. (Resp_12)

This statement reveals missed opportunities for intergenerational collaboration. Youth engagement could enhance physical maintenance capacity, innovation, and long-term stewardship. Their exclusion represents a lost resource in strengthening sustainability and community resilience. Ultimately, ownership emerged as the defining element linking social trust and health protection. A water committee member stated:

When the community feels ownership, they protect the borehole. (Resp_03)

Ownership transforms infrastructure from an externally delivered project into a collectively valued asset. When communities internalise responsibility, they actively safeguard facilities, discourage misuse, and maintain hygienic conditions. This sense of stewardship directly supports disease prevention and reinforces sustainable service delivery.

The findings reveal that meaningful participation, transparency, and inclusive decision-making strengthen community ownership, trust, and maintenance commitment, linking governance quality to WASH sustainability. Conversely, exclusion and weak accountability undermine collective action, service continuity, and preventive hygiene practices, increasing health risks. Sustaining WASH services therefore requires social cohesion, accountable governance, and trust-based community participation.

### Theme 5: environmental stress and system fragility

Environmental pressures, including drought, heavy rainfall, seasonal variability, and climate change, intensified W&S sustainability challenges by disrupting water availability, sanitation facilities, and community health. These pressures interacted with ageing infrastructure and weak governance, exposing deficiencies in project planning and management. As one respondent explained,

During drought, the borehole yield reduces and queues become very long. (Resp_06)

Reduced borehole output during dry periods creates scarcity that extends beyond inconvenience. Long queues increase time burdens especially for women and children, and may drive households to seek alternative, often unsafe, water sources. Scarcity also limits water use for hygiene, thereby weakening disease prevention practices such as handwashing and cleaning. Heavy rainfall presented a different but equally destabilising challenge. Another participant explained:

Heavy rains flood latrines and contaminate nearby water sources. (Resp_18)

This statement illustrates the interconnectedness of sanitation and water systems. Flooding compromises pit latrines, allowing faecal matter to contaminate surrounding soils and shallow water points. In poorly planned or low-lying areas, extreme rainfall transforms sanitation infrastructure into contamination pathways, undermining both environmental and public health safeguards. Perceived water quality deterioration during rainy periods further influenced behaviour. A caregiver stated:

When water smells after rains, people fear drinking it, but they still do. (Resp_09)

This quote reveals the tension between risk awareness and limited alternatives. Even when sensory cues suggest contamination, households continue using available sources because safer options are absent. Environmental shocks therefore expose not only technical vulnerabilities but also constrained decision-making under scarcity. Health workers observed seasonal disease patterns linked to environmental variability. A VHT member noted:

We see more diarrhoea cases during rainy seasons. (Resp_15)

This observation reinforces the link between climate-related disruptions and waterborne disease incidence. Rainfall-induced contamination and flooding appear to correlate with increased gastrointestinal infections, demonstrating how environmental stress translates directly into measurable health outcomes when sustainability systems are fragile. Long-term climatic shifts were also acknowledged. An elder reflected:

Climate is not like before. Wells dry faster now. (Resp_17)

This perception of changing environmental patterns suggests declining groundwater reliability and greater unpredictability in water supply. Such shifts demand adaptive planning, yet many systems appear designed for historical climatic conditions rather than current variability. Local leadership capacity to manage environmental stress was described as limited. A local leader explained:

We were not trained to manage these changing conditions. (Resp_02)

This statement highlights the governance dimension of environmental fragility. Without technical training, climate adaptation planning, or early warning systems, local actors struggle to respond effectively to droughts and floods. Environmental variability thus exposes institutional gaps in preparedness and adaptive management. Participants summarised the cumulative effect succinctly. One respondent concluded:

Environmental problems make already weak systems worse. (Resp_08)

This observation captures the systemic nature of vulnerability. Environmental stress does not create fragility in isolation; rather, it magnifies pre-existing governance weaknesses, technical deficiencies, and financial constraints. Where systems are already strained, climatic pressures accelerate deterioration and heighten exposure to disease.

The findings reveal that droughts, floods, and climate variability amplify technical, financial, and governance weaknesses, disrupting WASH services and increasing disease risks. Poor maintenance, weak institutional preparedness, and limited participation intensify unsafe coping behaviours and health vulnerabilities. Thus, WASH sustainability is a multidimensional resilience process requiring climate-responsive governance, adaptive planning, accountable leadership, and community engagement to advance SDG 6 and protect SDG 3 health outcomes.

## Discussion

This study examined how W&S project sustainability influences community health outcomes in Butambala District, Uganda. The findings reveal that infrastructure failures, delayed maintenance, weak governance, and limited participation disrupt service continuity, forcing households to adopt unsafe coping practices that increase disease vulnerability. These findings align with evidence linking inadequate WASH services to preventable morbidity, particularly diarrhoeal diseases (Prüss-Ustün *et al.*, 2019; Wolf *et al.*, 2018). They further demonstrate that sustainability is essential for translating SDG 6 investments into sustained SDG 3 health benefits.

Infrastructure reliability emerged as a critical determinant of health protection. Borehole breakdowns, deteriorating sanitation facilities, and delayed repairs compelled households to use unsafe water sources and reduce hygiene practices. This supports evidence that reliable WASH services reduce diarrhoeal risks more effectively than intermittent or inadequate provision (Wolf *et al.*, 2018; Fewtrell *et al.*, 2005). However, the findings extend this evidence by illustrating how service interruptions trigger risky behavioural adaptations, including reliance on contaminated sources and reduced handwashing. Consistent with Montgomery *et al.* (2009) and Lockwood and Smits (2011), these experiences demonstrate that infrastructure functionality depends on preventive maintenance, spare-part availability, and sustained institutional support rather than construction alone.

Household coping strategies further revealed how sustainability failures translate into health risks through constrained decision-making. Participants described water rationing, prolonged storage, and reliance on unsafe alternatives as responses to unreliable services. Although these practices reflect community resilience, they may compromise hygiene and increase disease exposure. While existing research establishes the health benefits of improved WASH services (Wolf *et al.*, 2018; Fewtrell *et al.*, 2005), the present study provides qualitative insight into how service unreliability shapes everyday hygiene decisions, demonstrating that physical access alone cannot guarantee sustained health improvements.

Governance emerged as another critical mechanism influencing sustainability. Unclear institutional responsibilities, weak accountability, financial opacity, and fragmented coordination delayed maintenance and undermined community confidence. These findings support Whaley and Cleaver's (2017) argument that technical functionality cannot be separated from institutional and governance dynamics. Similarly, Chowns (2015) attributes rural water failures to systemic governance weaknesses rather than community unwillingness. The Butambala findings extend these perspectives by demonstrating how governance deficiencies disrupt service continuity and indirectly increase disease vulnerability. This reinforces Lockwood and Smits' (2011) emphasis on structured post-construction support and sustained coordination between communities and district authorities.

Social trust and meaningful participation also influenced sustainability outcomes. Transparent leadership and inclusive decision-making encouraged community contributions and maintenance commitment, while exclusion, financial mistrust, and declining participation weakened collective responsibility. These findings align with Mansuri and Rao (2013), who emphasise accountability and institutional inclusion in participatory development, and Montgomery *et al.* (2009), who recognise social dimensions of WASH sustainability. Gender-related participation inequalities further demonstrate that community ownership depends on equitable involvement and institutional legitimacy rather than formal committee structures alone (Whaley and Cleaver, 2017).

Environmental pressures, particularly droughts, heavy rainfall, and climate variability, further intensified infrastructure and governance weaknesses. These conditions disrupted water availability, increased contamination risks, and exposed limitations in maintenance preparedness. Rather than operating independently, environmental shocks interacted with financial constraints, weak coordination, and unreliable infrastructure, reinforcing the multidimensional nature of sustainability. This finding highlights the importance of climate-responsive planning and institutional resilience in sustaining WASH services.

Overall, the findings demonstrate that WASH sustainability depends on interconnected technical, financial, governance, social, and environmental factors that shape community health outcomes. Sustained health benefits require reliable infrastructure, accountable leadership, effective maintenance, and meaningful participation. Thus, sustainability provides the critical link between SDG 6 investments and SDG 3 health outcomes in rural Uganda.

## Conclusion

This study examined how the sustainability of W&S projects influences community health outcomes in Butambala District, Uganda. The findings demonstrate that sustainability is not simply a technical or operational matter but a structural determinant of public health. When infrastructure breaks down, maintenance is delayed, governance is weak, and accountability is unclear, households resort to unsafe water sources and compromised hygiene practices, increasing exposure to disease. Conversely, where leadership is responsive, roles are clearly defined, and maintenance systems function effectively, W&S projects serve as protective assets that sustain health gains. The study therefore clarifies that achieving SDG 6 in rural contexts requires more than infrastructure expansion; it demands sustained service delivery systems that safeguard SDG 3 health outcomes over time.

### Policy, practice, societal, and theoretical implications

#### Policy and practice implications

The findings underscore the need for Uganda's WASH policies to shift from infrastructure expansion toward sustainable service delivery. Despite improvements in W&S access, persistent functionality and maintenance challenges require stronger post-construction management, preventive maintenance, adequate financing, clearly defined institutional responsibilities, and continuous technical support for community-managed systems.

At the district level, coordination among District Water Offices, water user committees, and VHTs should be strengthened by integrating WASH monitoring into routine community health surveillance. Early detection and timely repair of infrastructure failures would reduce reliance on unsafe alternatives and associated disease risks, reinforcing SDG 6–SDG 3 integration. Consistent with Uganda's decentralisation framework, transparent financial reporting, inclusive decision-making, and clear accountability among community leaders, local authorities, and technical personnel are essential for strengthening trust, participation, and service continuity.

Furthermore, gender-responsive governance and climate-resilient planning should guide WASH investments. Women, who often bear primary responsibility for water collection and household hygiene, should have greater representation in management and financial decisions. Infrastructure planning should also address droughts, floods, and rainfall variability through adaptive maintenance and preparedness strategies. These measures would strengthen resilience, reduce repeated rehabilitation costs, improve investment efficiency, and sustain long-term community health benefits.

#### Implications for society and community well-being

The findings demonstrate that sustainable WASH systems have direct implications for community quality of life, dignity, and health security. When W&S services fail, households are forced to make difficult trade-offs that affect hygiene practices, disease prevention, and daily productivity. Improving sustainability therefore contributes not only to reducing disease exposure but also to reducing household burdens, particularly among women and children who are often responsible for water-related activities.

The study further suggests that public attitudes toward WASH projects are shaped by trust and perceived reliability. Communities are more likely to protect and contribute to infrastructure when they experience transparency, responsiveness, and meaningful participation. Therefore, strengthening community ownership should involve genuine inclusion rather than transferring responsibility without adequate resources or institutional support.

#### Theoretical and research implications

The study advances WASH sustainability scholarship by conceptualising sustainability as a multidimensional, dynamic process linking infrastructure investments to community health outcomes. Rather than treating functionality as the primary indicator, the findings demonstrate that service continuity depends on interactions among technical, governance, economic, social, and environmental conditions. This perspective explains how institutional arrangements influence whether infrastructure investments translate into sustained health benefits.

The findings further extend CBM perspectives by demonstrating that participation alone is insufficient without accountable governance, technical capacity, financial support, and sustained institutional engagement. This provides a context-sensitive explanation for variations in sustainability outcomes across communities. Additionally, the study contributes to SDG integration scholarship by identifying the sustainability mechanisms through which SDG 6 investments support SDG 3 health outcomes, demonstrating that this relationship is conditional rather than automatic. Overall, the study strengthens theoretical understanding of WASH sustainability as an integrated process connecting governance, community participation, financial viability, environmental resilience, and public health protection.

## Limitations and future research

This study has several limitations. First, its qualitative case study design, confined to Butambala District, limits the generalisability of findings, while the absence of quantitative health surveillance data prevents establishing measurable associations between project sustainability and disease incidence. Second, reliance on self-reported experiences introduces potential recall bias and subjective interpretations of health outcomes. Third, the cross-sectional design captures experiences at a single point in time, limiting understanding of seasonal variations, climatic changes, and governance transitions. Although multiple stakeholders participated, perspectives from higher administrative levels may not have been fully represented. Future research should employ longitudinal and mixed-method designs integrating qualitative evidence with epidemiological and infrastructure functionality data to examine sustainability–health relationships over time. Comparative district-level studies could identify effective governance and service delivery models across rural contexts. Further investigation into climate variability, gendered governance dynamics, and institutional accountability would deepen understanding of sustainability–health linkages and inform integrated SDG 6 and SDG 3 implementation strategies in rural Sub-Saharan Africa.
